# Neisseria gonorrhoeae PIII has a role on NG1873 outer membrane localization and is involved in bacterial adhesion to human cervical and urethral epithelial cells

**DOI:** 10.1186/1471-2180-13-251

**Published:** 2013-11-09

**Authors:** Rosanna Leuzzi, Barbara Nesta, Elisabetta Monaci, Elena Cartocci, Laura Serino, Marco Soriani, Rino Rappuoli, Mariagrazia Pizza

**Affiliations:** 1 Novartis Vaccines and Diagnostics, S.r.L, Via Fiorentina 1, Siena 53100, Italy

**Keywords:** *Neisseria gonorrhoeae*, PIII, OmpA, adhesion, Outer membrane, RmpM homologue

## Abstract

**Background:**

Protein PIII is one of the major outer membrane proteins of *Neisseria gonorrhoeae*, 95% identical to RmpM (reduction modifiable protein M) or class 4 protein of *Neisseria meningitidis*. RmpM is known to be a membrane protein associated by non-covalent bonds to the peptidoglycan layer and interacting with PorA/PorB porin complexes resulting in the stabilization of the bacterial membrane. The C-terminal domain of PIII (and RmpM) is highly homologous to members of the OmpA family, known to have a role in adhesion/invasion in many bacterial species. The contribution of PIII in the membrane architecture and its role in the interaction with epithelial cells has never been investigated.

**Results:**

We generated a *ΔpIII* knock-out mutant strain and evaluated the effects of the loss of PIII expression on bacterial morphology and on outer membrane composition. Deletion of the *pIII* gene does not cause any alteration in bacterial morphology or sensitivity to detergents. Moreover, the expression profile of the main membrane proteins remains the same for the wild-type and knock-out strains, with the exception of the NG1873 which is not exported to the outer membrane and accumulates in the inner membrane in the *ΔpIII* knock-out mutant strain.

We also show that purified PIII protein is able to bind human cervical and urethral cells and that the *ΔpIII* knock-out mutant strain has a lower ability to adhere to human cervical and urethral cells.

**Conclusion:**

Here we demonstrated that the PIII protein does not play a key structural role in the membrane organization of gonococcus and does not induce major effects on the expression of the main outer membrane proteins. However, in the PIII knock-out strain, the NG1873 protein is not localized in the outer membrane as it is in the wild-type strain suggesting a possible interaction of PIII with NG1873. The evidence that PIII binds to human epithelial cells derived from the female and male genital tract highlights a possible role of PIII in the virulence of gonococcus and suggests that the structural homology to OmpA is conserved also at functional level.

## Background

The three major outer membrane proteins of *N. gonorrhoeae* have been historically denoted as protein I, II and III (PI, PII and PIII) [1,2], with PIII forming a trimer with two molecules of PI [2]; PI and PII have been subsequently described as porin and Opa proteins, respectively [3-5]. It is worth noting that, in contrast to PorB and Opa, which undergo, respectively, antigenic and phase variation, PIII is highly conserved and expressed by all pathogenic Neisseriae [6]. This characteristic inter- and intra-strain homogeneity is unique among all the main outer membrane constituents, which, contrary to PIII, evolved a strong variability to escape the immune pressure of the host [7,8].

PIII has been mainly studied for its peculiarity to induce “blocking antibodies” able to prevent the formation of the lytic complement attack complex and blocking the bactericidal activity of antibodies raised against other surface antigens [9,10].

The ability to construct a viable gonococcal mutant lacking the *pIII* gene was described by Wetzler and collaborators in 1989. In that study the F62 *Neisseria gonorrhoeae* strain knocked-out for the *pIII* gene resulted to be identical to the wild-type strain in terms of competence, porin activity, protease and antibiotic sensitivity. The mutant had minimal differences in colony morphology and was slightly decreased in growth compared to the parent strain [11].

PIII is 95% identical to class 4 protein of *Neisseria meningitidis*, also named RmpM (reduction modifiable protein M) for the characteristic migration in SDS-PAGE in presence of reducing agents [12]. The presence of RmpM in oligomeric complexes of the outer membrane has been extensively described, with RmpM transiently associated to the porins, depending on the specific transport needs during the different stages of meningococcal life cycle [13,14]. Moreover, RmpM forms heterooligomeric complexes with iron limitation-inducible OMPs [15] and associates through the N-terminal domain to the Omp85 complex [16].

The C-terminal region of PIII shows high similarity to the outer membrane protein A (OmpA) of *E. coli* and other Gram-negative bacteria [17]. OmpA has been studied in *E. coli* as a key factor in many pathogenicity processes. The expression of OmpA contributes to the structural integrity of the outer membrane [18] and confers a significant selective advantage during the pathogenesis *in vivo*; an *ompA* mutant showed indeed an attenuated virulence in two different models of *E. coli* K1 infection and increased sensitivity to serum bactericidal activity [19].

The crystal structure of the OmpA-like domain of the meningococcal RmpM has been solved [20] revealing the presence of a C-terminal peptidoglycan-binding domain, which could stabilize the neisserial outer membranes promoting the tight interaction between the outer membrane and the peptidoglycan layer.

To further expand the findings of Wetzler et al. [11] and unravel the role of PIII in the physiology of gonococci, we applied microscopy and biochemical approaches. Although we excluded a direct role of PIII in maintaining the structural integrity of gonococcus, we observed that the lack of PIII affects the translocation of the NG1873 protein, with an unknown function, to the outer membrane, opening new insights on the role of this protein in gonococcal physiology and on the significance of PIII-NG1873 interaction.

Moreover, to study the biological implication of the presence of the OmpA-like domain we tested the ability of PIII to mediate adhesion to epithelial cells and we showed that PIII facilitates bacterial adhesion to human epithelial cells derived from the female and male genital tracts suggesting a possible role in gonococcal colonization.

## Results

### Lack of PIII has no effect on bacterial shape and membrane perturbation

**Figure 1:**
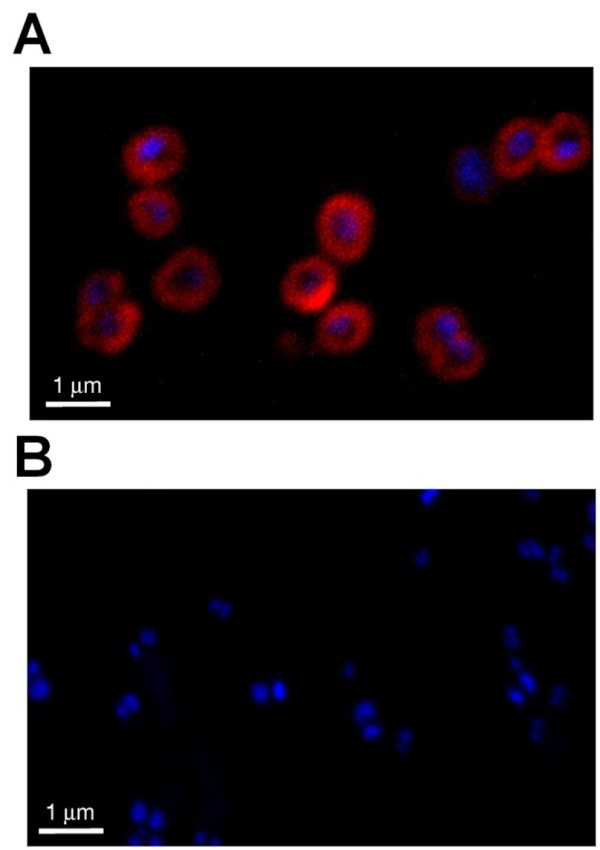
**Localization of pIII protein on the surface of F62 strains.** Confocal microscopy analysis of F62 wild-type **(A)** and F62Δ*pIII* knock-out strains **(B)**. DNA was stained with DAPI (blue) whereas PIII protein was labeled with mouse anti-PIII antibodies, followed by Alexa Fluor 568 dye antibody (red).

To investigate the role of PIII in the physiology of *N. gonorrhoeae*, an F62Δ*pIII* isogenic mutant was generated by replacing the *pIII* gene with an erythromycin resistance cassette. Lack of PIII expression in F62Δ*pIII* strain was verified by Western blot analysis on whole cell extract (data not shown) and by confocal microscopy with mouse anti-PIII polyclonal antibodies. The results, reported in Figure 1A, show that PIII is widely distributed on the F62 bacterial surface. As expected, no membrane staining was observed in the F62Δ*pIII* mutant strain (Figure 1B).

**Figure 2:**
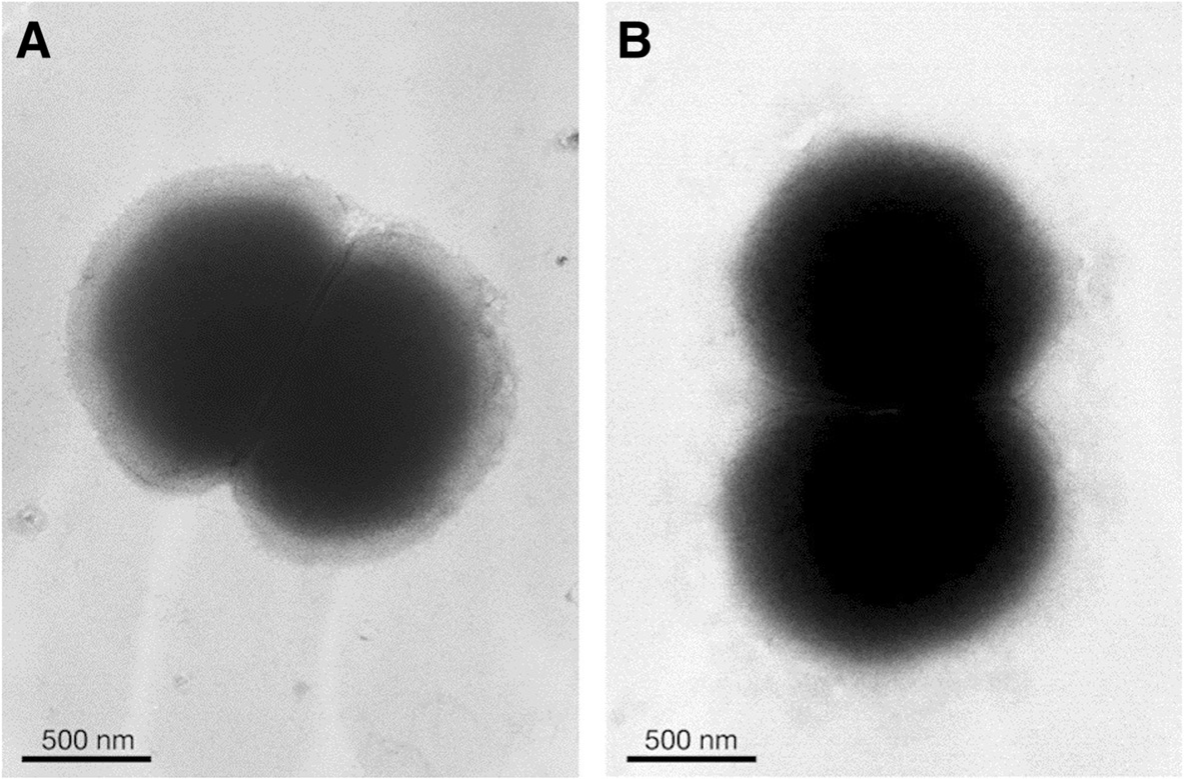
**Negative staining and TEM analysis of F62 wild-type (A) and F62Δ*pIII *(B) strains.** The sizes of diplococci from the wild type and mutant strains are 2.296 ± 0.0819 μM and 2.275 ± 0.075 μM, respectively. Values are the mean ± SEM from 20 images for each strain.

Transmission electron microscopy by negative staining of the wild type F62 versus the F62Δ*pIII* mutant strain shows that absence of PIII protein does not cause any alteration in bacterial size and shape (Figure 2). Moreover, sensitivity to detergent like SDS, Triton X-100 and deoxycholate, tested by paper disk diffusion inhibiting assays, was identical for the two strains. The MICs (minimal inhibitory concentrations) were 0.12% for SDS, 0.06% for Triton X-100 and 0.03% for deoxycholate for both, wild- type and knock-out strains confirming the hypothesis that the loss of PIII does not induce any perturbation in membrane resistance and/or membrane structure.

### Lack of PIII does not alter the expression of the main membrane proteins but influences the membrane localization of NG1873

Since the meningococcal orthologous of PIII, RmpM, is part of heterooligomeric complexes of the outer membrane with a possible stabilizing function on meningococcal membrane [14-16,21], we verified whether the deletion of the *pIII* gene causes any alteration on outer membrane composition.

**Figure 3:**
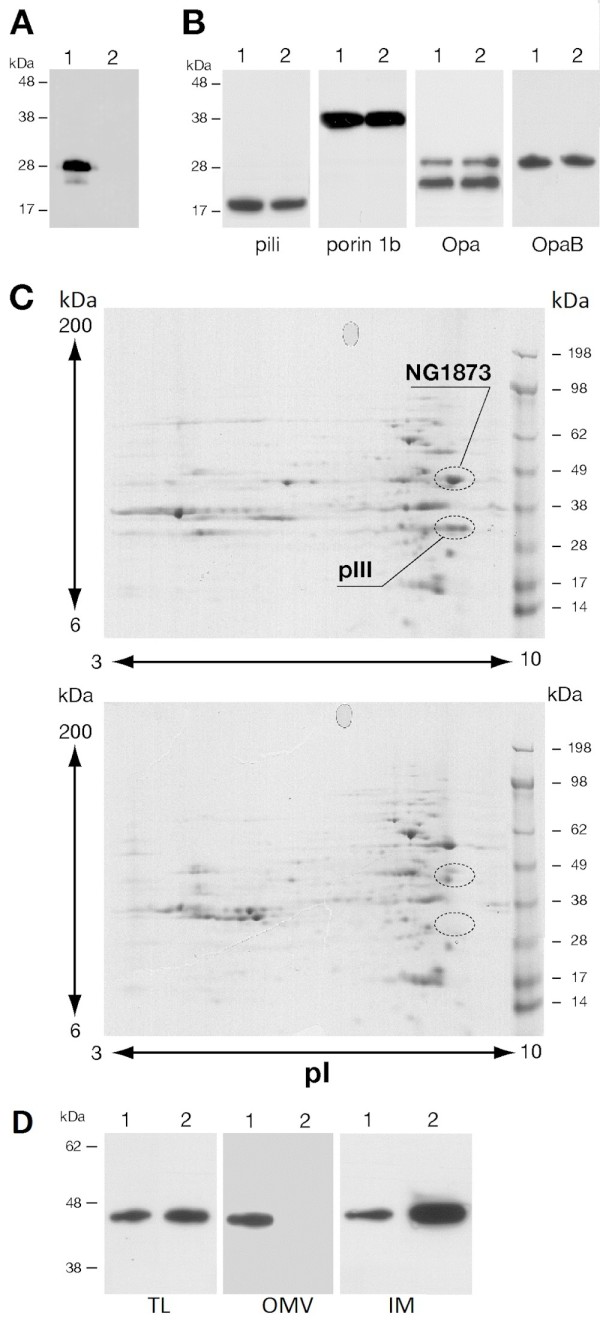
**Characterization of OM prepared from F62 wild-type and F62Δ*pIII *strains. A**. Western blot analysis of OM prepared from F62 wild-type (lane 1) and F62Δ*pIII* strains (lane 2) using mouse anti-PIII serum. **B**. Expression of the main component of the gonococcal OM prepared from F62 wild-type (lane 1) and F62Δ*pIII* strains (lane 2); specific antibodies against each protein were used. **C**. 2-DE of OM prepared from F62 wild-type (upper panel) and F62Δ*pIII* strains (lower panel). The PIII protein and the protein encoded by the gene *ng1873* are shown in circled spots. **D**. Western blot analysis of total lysates (TL), outer membranes (OM) and inner membranes (IM) from F62 wild-type (lane 1) and F62Δ*pIII* strains (lane 2) using mouse anti-NG1873 serum.

Western blot analysis on outer membranes (OM) confirmed the absence of the PIII protein in the mutant strain (Figure 3A) and showed that the levels of expression of pili, porin 1b, Opa proteins and OpaB variant were unchanged in F62Δ*pIII* strain compared to the wild-type (Figure 3B).

To explore in more detail the composition of the outer membrane, OM deriving from the wild-type and the Δ*pIII* strains were analyzed by 2D electrophoresis (Figure 3C). By comparative analysis of the 2D electrophoresis maps, only two proteins appeared to be differentially expressed in the OM deriving from the wild-type (upper panel) and absent in the OM deriving from the Δ*pIII* strain (lower panel). The two spots (circled in Figure 3C) were identified by mass spectrometry and shown to be the protein PIII and the protein encoded by the *ng1873* gene. Western blot analysis with mouse anti-NG1873 polyclonal antibodies showed that while the level of expression of NG1873 in total cell lysates from the wild-type and the Δ*pIII* mutant strains was comparable, the protein was not detected in the OM from the Δ*pIII* mutant strain (Figure 3D). Interestingly, the amount of NG1873 was significantly higher in the inner membranes deriving from the Δ*pIII* mutant strain (Figure 3D) suggesting that the lack of the PIII protein causes a defective outer-membrane localization of NG1873 protein and its accumulation in the inner membrane.

### Purified PIII is able to bind to human immortalized cervical and urethral cell

The C-terminal domain of PIII shows significant homology to OmpA proteins described in other microorganisms and known to mediate adhesion to eukaryotic cells, with identities and similarities ranging from 35 to 45% and from 50 to 60%, respectively. To verify whether the sequence similarity to OmpA was representative also of a functional homology, we tested the ability of PIII to bind epithelial cells. To this aim, the recombinant PIII protein (devoid of the signal peptide) was expressed in *E. coli*, purified from the cytoplasm in its soluble form and tested in the adhesion assay. As cell models we used three immortalized human epithelial cell lines derived from primary ectocervical, endocervical and urethral cells which maintained all main features of primary cells [22,23].

**Figure 4:**
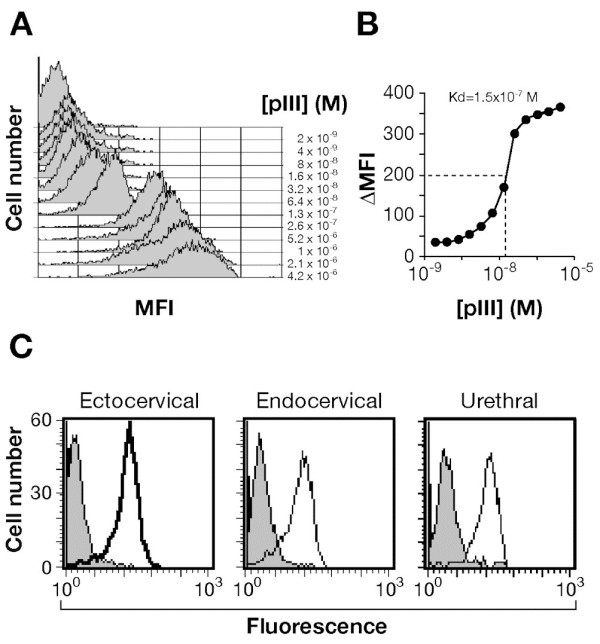
**Binding of purified recombinant PIII protein to epithelial cells. A**. Ectocervical cells were incubated for 1 h at 37°C with increasing concentrations of the purified PIII protein (range 2 nM-4.2 μM). The binding was analysed by FACS using mouse anti-PIII antibodies and an R-Phycoerythrin-conjugated secondary antibody. The values are reported as net mean fluorescence intensity (MFI). **B**. Saturation curve of PIII binding to ectocervical cells. Analysis was performed on data reported in A. The *K*_d_ value was calculated as the PIII concentration that determines the saturation of 50% of the receptors present on the cells. **C**. Representative flow cytometry profiles of the binding of 1 μM PIII to ectocervical, endocervical and urethral cells. Grey line profiles represent the cells incubated with the primary and secondary antibodies in absence of the PIII protein.

Cells were incubated with increasing amount of the purified PIII protein and binding measured by FACS analysis. The PIII protein binds all the cell lines tested. As shown in Figure 4A, the PIII binding to ectocervical cells increased in a dose-dependent manner reaching a plateau at concentration of 1 μM. The affinity of the PIII binding was determined by plotting the mean fluorescence intensity versus the protein concentration. The K_d_ value, defined as PIII concentration able to saturate 50% of putative receptors, was estimated in the order of 1.5×10^-7^ M (Figure 4B). The binding of PIII protein to endocervical and urethral cells had a similar trend, showing the higher degree of association at 1 μM (Figure 4C). The unrelated hypothetical protein NG0694 of *N. gonorrhoeae*, used as negative control in the assay, was unable to bind all the cell lines tested (data not shown).

### PIII is involved in adhesion of *N. gonorrhoeae* to human immortalized cervical and urethral cells

**Figure 5:**
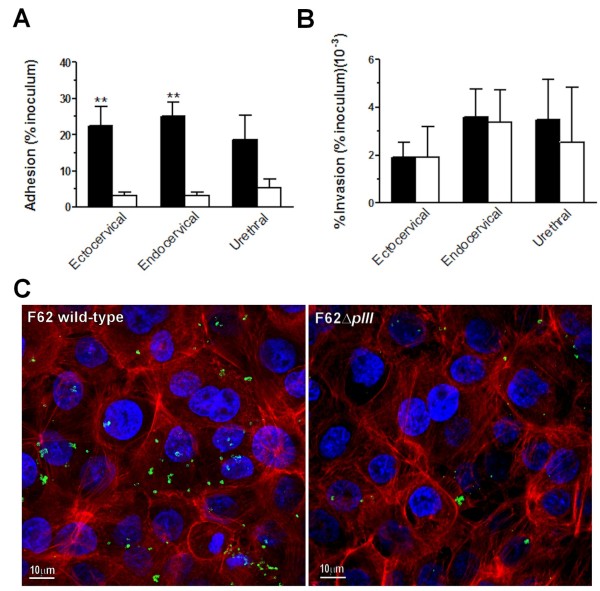
**Adhesion (A) and invasion (B) of F62 wild-type (black columns) and F62Δ*pIII *(white columns) strains to ectocervical, endocervical and urethral cells.** Cells were infected for 3 hours at an MOI of 100:1. Total cell-associated bacteria and intracellular gentamicin-resistant bacteria were quantified after lysis of cell membranes with 1% saponin followed by dilution and plating. In competition experiments, ectocervical cells were pre-incubated with 25 μg/mL of pIII protein before infection (grey column). Results are means ± SEM from three independent experiments, each performed in triplicate. The high variability in the values shown in the Figure 3B was due to the very low number of the intracellular bacteria. ** p < 0.01. **C**. Ectocervical cells were infected for 3 hours with F62 wild-type (left panel) and F62Δ*pIII* (right panel) strains and, after washing, were fixed and stained for confocal immunoflurescent microscopy. Bacteria were labeled by an anti-OM serum and a secondary fluorescent antibody (green). DNA and cellular actin were stained with DAPI (blue) and Phalloidin-Alexa Fluor 568 (red), respectively.

To verify whether the ability of PIII to bind epithelial cells as purified protein was relevant also in the context of the viable microorganism, we performed infection assays and compared the ability of the F62 wild-type and the F62Δ*pIII* strains to adhere to ectocervical, endocervical and urethral cells previously described. Cells were infected with wild-type and F62Δ*pIII* strains for 3 hours and, after cellular lysis, total cell-associated bacteria were counted by plating. Since the level of gonococcal invasion is very low in piliated strains, the number of total bacteria collected was considered to be representative of the number of bacteria adhering to the cell surface. Results reported in Figure 5A, show a decrease in bacterial association to all three epithelial cell lines for the *pIII*-deficient strain with a more pronounced effect on cervical cells (≈ 6–8 fold reduction) than on urethral cells (2.5-fold reduction). These data were confirmed by immunofluorescence confocal microscopy analysis, showing a larger number wild-type bacteria associated to ectocervical cells compared to Δ*pIII* strain (Figure 5C).

Influence of PIII in invasion was evaluated by plating the intracellular bacteria recovered following gentamycin killing of extracellular bacteria. As expected only a low percentage of gonococci were able to invade epithelial cells; levels of invasion were similar for the wild-type F62 and *ΔpIII* mutant strains (Figure 5B).

To exclude that differences in adhesion could be due to a defect of growth of the Δ*pIII* mutant strain [11], the growth rate of both strains in the cell culture medium was monitored during the time of infection. The growth rate of gonococci in the cell culture medium was very low but identical for the two strains (data not shown). Moreover, expression of phase-variable Opa proteins and pili, the structures known to be the main factors involved in the adhesion to epithelial cells, were analyzed by Western Blot. The wild-type and the *ΔpIII* mutant strains used in this study are piliated and express similar amounts of Opa proteins (data not shown).

The impaired ability of the Δ*pIII* mutant strain to bind to the epithelial cells was not due to the absence of NG1873 on the outer membrane, since the knock-out Δ*ng1873* mutant strain had an adhesive phenotype on ectocervical cells comparable to the wild-type strain (data not shown).

## Discussion

PIII is one of the main components of the outer membrane of Neisseria, but its precise function, both in the pathogenesis and in the physiology of the organism, remains unclear. In an effort to better define the role of PIII in gonococcus, we generated a knock-out Δ*pIII* F62 strain and investigated the impact of this deletion on bacterial cell morphology and adhesion.

A mutant F62 strain lacking the PIII protein in *N. gonorrhoeae* was previously described showing no severe defects compared to the wild type strain in terms of competence, porin activity, protease and antibiotic sensitivity. The mutant had minimal differences in colony morphology and was slightly decreased in growth compared to the parent strain [11].

Here we have expanded the finding of Wetzler’s study showing that the lack of the PIII protein in the *pIII*-knock-out strain does not influence the bacterial shape or resistance to detergents corroborating the hypothesis that PIII does not play a key role in the organization of the outer membrane. In the *pIII*-mutant strain, the only clear difference in 2D gel analysis was a defective localization for the NG1873 protein. Interestingly, NG1873 has a LysM domain (in position 35–83), with a peptidoglycan binding function [24]. We can speculate that NG1873 is able to reach the outer membrane only when PIII is acting as a bridge between the outer membrane and the peptidoglycan layer. Further studies will be needed to evaluate the role of this interaction in the context of peptidoglycan and outer membrane architecture and to explore the involvement of other proteins in the NG1873 bacterial localization.

The crystal structure of the C - terminal domain of the meningococcal RmpM has been solved [20]. The authors have identified a number of residues which may participate in the non-covalent binding of peptidoglycan. They envisage a model in which the C-terminal domain RmpM interacts with peptidoglycan stabilizing oligomers of porins in the outer membrane. Since the peptidoglycan of Gram-negative bacteria is located in the periplasmic space, this model in combination with the evidence that the N-terminal part of RmpM is associated to the OMP complexes but is too short to cross the membrane [16], would imply a periplasmic localization for the entire protein. However the proposed periplasmic localization is not supported by the evidence that the RmpM/PIII protein is an immuno-dominant antigen with surface-exposed epitopes [1]. In this study we confirmed the surface exposure of PIII by confocal microscopy analysis.

The similarity between PIII and proteins belonging to the OmpA family, known to have a role in adhesion in many bacterial species, has driven the investigation on the potential contribution of PIII in adhesion process. Here we provide evidences that gonococcal pIII mediates bacterial adhesion to human epithelial cells, derived from the female and male genital tracts.

The choice of a cellular model to study factors and mechanisms involved in the gonococcal pathogenesis is a crucial topic of debate. The data obtained from *in vitro* models of infection can lead to conclusions not fully relevant with respect to the natural infection. In fact, whereas by the bacterial side, gonococcus undergoes antigenic and phase variation depending on the particular selective pressure induced by cellular contact, by the cellular side, the cell lines can substantially differ from the parental tissue in terms of membrane receptors and functional responses. Although the relevance of any model of infection is not exactly predictable, we limited the possible biases by examining the expression of pili and Opa proteins in the wild-type and *pIII*-deficient strains used in the infection assays. Moreover, to simulate the female and male infection, we used primary immortalized cell lines obtained from ectocervix, endocervix and urethra. All of three immortalized cells have been characterized to ensure that they maintain the main features of the primary cells [22,23]. These cellular systems allowed to overcome the problems of limited life span and limited number of primary cells deriving from surgical tissues; moreover, it is a better model respect to the cancer-derived cell lines which can strongly differ from *in vivo* tissues. In our studies we show that the *pIII*-deficient strain has an impaired ability to associate to cervical cells and, to a lesser extent, to urethral cells. These observations, together with the evidence that the purified PIII protein is able to specifically bind to all the three cell lines, support the hypothesis that PIII could have a role in gonococcal colonization of the genital tract. The impaired adhesive phenotype was not a secondary effect of the outer membrane reorganization since we demonstrated that deletion of the *pIII* gene has no major effects on the expression of the main outer membrane proteins.

We previously described an OmpA-like protein in gonococcus, denoted as Ng-OmpA [25] which plays a significant role in the adhesion and invasion processes into human cervical and endometrial cells. These results suggest that the OmpA domain has a redundant function in gonococcus and that it could have a role at different stages of infection; however, additional studies will be needed to explore the respective role of these two proteins in gonococcal pathogenesis.

## Conclusions

In conclusion, we demonstrated that PIII protein of *N. gonorrhoeae* does not influence the outer membrane integrity as well as bacterial shape, morphology and strain sensitivity to detergents. However, the loss of expression of PIII protein causes a defective membrane localization of NG1873, a protein having a LysM domain with a putative peptidoglycan binding function. Our study also demonstrated that PIII has a role in the interaction with human cervical and urethral cells, suggesting an involvement in the gonococcal adhesion process.

## Methods

### Bacterial strains and growth conditions

*Neisseria gonorrhoeae* F62 strain was grown overnight in gonococcus medium (GC) agar (Difco) or in liquid GC broth supplemented with 1% isovitalex (BBL) at 37°C in 5% CO_2_.

### Cloning and construction of isogenic mutants

The *pIII* and *ng1873* genes devoid of the sequence for the predicted leader peptide (sequences coding for amino acids 1–22) and the stop codon were amplified using the primers FOR-pIII-5′-cgcggatcccatatg GGCGAGGCGTCCGTT-3′ (*Nde*I site), REV-pIII-5′-cccgctcgagGTGTTGGTGATGATTGCG-3′ (*Xho*I site), FOR-ng1873-5′-cgcggatcccatatgGCAAATCTGGAGGTGCGCC-3′ (*Nde*I site), REV-ng1873-5′-cccgctcgagTTGGAAAGGGTCGGAATCG-3′ (*Xho*I site). The PCR products were inserted into the *Nde*I/*Xho*I sites of the pET21b expression vector in order to obtain the pET-pIII-His and pET-ng1873-His constructs.

Knockout mutants in F62 strain, in which the *pIII* and the *ng1873* genes were truncated and replaced with an antibiotic cassette, were prepared as described in [25]. The gene deletion was verified by PCR and lack of protein expression was confirmed by Western blot analysis.

### Purification of recombinant His-tagged proteins and preparation of polyclonal antisera

PIII and NG1873 His-tagged proteins were expressed in *E. coli* as described [25] and proteins were purified on a metal-chelate affinity chromatography column (MCAC); proteins were eluted in a single step with 50 mM phosphate buffer pH 8.0, 300 mM NaCl, 250 mM imidazole and protease inhibitors.

To prepare antisera against PIII and NG1873 His-tagged proteins, 20 μg of each purified protein were used to immunize CD1 female mice. The recombinant proteins were given i.p. together with Al(OH)_3_ for three doses (day 0, day 21, day 35). Blood samples were taken on day 49 and pooled.

### Confocal immunofluorescence microscopy

To visualize PIII protein on bacterial surface, F62 strain was grown in GC up to OD_600_ 0.5 and washed in PBS. Bacterial pellet were fixed with 2% PFA for 20 min at room temperature and spotted on chamber slides coated with poly-lysine. Bacteria were then blocked with 2% BSA for 15 min and incubated with mouse polyclonal anti-PIII antibodies diluted in 2% BSA for 30 min at room temperature. Bacteria were then stained with goat anti-mouse Alexa Fluor 568 conjugated antibodies (Molecular Probes) for 20 min at room temperature. Labeled samples were mounted with ProLong® Gold antifade reagent with DAPI and analyzed with Zeiss LSM710 confocal microscope.

### Negative staining and TEM

A drop of a 10^9^ cfu/mL bacterial suspension in D-PBS was placed on Parafilm and bacteria were adsorbed for 15 min to formvar/carbon 200 mesh grids. Bacteria were fixed for 15 min with 2% p-formaldehyde and grids were then rinsed four times in PBS and air-dried. Grids were finally treated with uranyl acetate and examined by TEM GEOL 1200EX II transmission electron microscope.

### Paper disk diffusion inhibiting assays

F62 and F62Δ*pIII* strains were grown overnight on GC agar, suspended in D-PBS and adjusted to OD_600_ = 0.1 (≅10^8^ cfu/mL). An aliquot of 0.1 mL of the bacterial suspension was seeded on GC agar. 10 μL of the following detergents were applied to paper disk (Oxoid): SDS at 0.125, 0.25, 0.5, 1%, Triton X-100 at 0.03, 0.06, 0.125, 0.25% and deoxycolate at 0.8, 0.9, 1.2, 1.4%. Control disks with PBS were included in the assay. Disks were then placed on the GC agar inoculated with bacteria. All plates were incubated at 37°C in 5% CO_2_.

### Cell fractionation and protein analysis

Total cell lysates (TL), inner membranes (IM) and outer membrane (OM) were prepared from bacteria at exponential growth phase. Total cell lysates were obtained by three freezing-thawing cycles.

For IM and OM preparation, bacteria were sonicated, unbroken cells were removed by centrifugation and the supernatant centrifuged at 50000 × g for 90 min at 4°C. The pellet containing the membranes was incubated in 2% Sarkosyl in 20 mM Tris–HCl, pH 7.5 at room tempertaure for 20 min to solubilize the inner membranes. The suspension was centrifuged at 10000 × g for 20 min at 4°C and the supernatant was centrifuged overnight at 75000 × g at 4°C. The supernatant was collected as IM fraction and the pellet, containing the OM, was resuspended in 20 mM Tris–HCl, pH 7.5.

SDS-PAGE electrophoresis with NuPage 4-12% Bis-Tris gels (Invitrogen) and Western blot analysis were performed according to standard procedures.

Opa proteins were detected by monoclonal antibody 4B12, kindly provided by M. Achtman. Pili were detected by monoclonal antibody SM1, kindly provided by M. Virji. OpaB protein was detected by polyclonal antisera against NG0070 His-tagged protein; purification of the protein and mice immunization were performed as described before. Bands were visualized with Super Signal Chemiluminescent Substrate (PIERCE).

### Two-dimensional gel electrophoresis and image analysis

200 μg of proteins were precipitated with 0.015% sodium deoxycholate and 48% trichloroacetic acid and dissolved in 7 M urea, 2 M thiourea, 2% CHAPS, 2% ASB14, 1% DTT, 2 mM tributylphosphine, 20 mM Tris and 2% carrier ampholyte. Proteins were absorbed overnight onto Immobiline DryStrips (7 cm; pH-gradient 3*–*10 non linear) and the first dimension was run using a IPGphor Isoelectric Focusing Unit (Ge Healthcare), applying sequentially 150 V for 1 hour, 500 V for 35 min, 1000 V for 30 min, 2600 V for 10 min, 3500 V for 15 min, 4200 V for 15 min and finally 5000 V to reach 12kVh. For the second dimension, strips were equilibrated as described and proteins were separated on linear 4*–*12% polyacrylamide gels. Bidimensional gel was acquired with a Personal Densitometer SI (Molecular Dynamics) and images were analyzed with the software Image Master 2D v2003.02 (Ge Healthcare).

### In-gel protein digestion and MALDI-TOF mass spectrometry analysis

Protein spots were excised from the gels, washed with 50 mM ammonium bicarbonate/acetonitrile 50/50 (v/v) and air-dried. Dried spots were digested for 2 hours at 37°C with sequencing grade modified trypsin in 5 mM ammonium bicarbonate, loaded on a matrix prespotted Anchorchip (PAC 384 HCCA, Bruker-Daltonics, Bremen, Germany), air-dried and washed with 70% ethanol, 0.1% trifluoracetic acid. Mass spectra were acquired on an ultraflex MALDI TOF mass spectrometer (Bruker-Daltonics). Spectra were externally calibrated by using the combination of standards present on the PAC (Bruker-Daltonics). Monoisotopic peptide matching and protein search were performed automatically by MASCOT software.

### Cell culture

Ectocervical and Endocervical cells (Ect1/E6E7 and End1/E6E7 from ATCC) were maintained in keratinocyte serum-free medium (KSFM, Gibco) supplemented with 50 μg/mL bovine pituitary extract, 0.1 ng/mL epidermal growth factor, 0.4 mM CaCl_2_ and antibiotics at 37°C in 5% CO_2_.

Transformed urethral epithelial cells (kindly provided by M. Apicella, Department of Microbiology, University of Iowa) were maintained in Prostate Epithelial Cell Growth Medium (PrEGM, Clonetics) supplemented with 10% heat-inactivated fetal bovine serum, antibiotics and growth factors included in PrEGM BulletKit at 37°C in 5% CO_2_.

### Binding assay and FACS analysis

Cells were non-enzymatically detached using cell dissociation solution (CDS, Sigma), harvested and suspended in RPMI medium supplemented with 1% FBS. Approximately 10^5^ cells were placed in 96-well microplates and mixed with different concentrations of purified PIII and NG0694 (negative control) proteins or medium alone for 1 hour at 37°C, mixing every 20 min to avoid the attachment of cells.

Excess unbound proteins were removed by two washings and centrifugations and cells were incubated for 1 hour at 4°C with anti-PIII and anti- NG0694 antisera followed by incubation with R-Phycoerythrin-conjugated anti-mouse IgG for 30 min at 4°C.

Cell-bound fluorescence was analysed with FACSCalibur flow cytometer (Becton Dickinson) by using the CellQuest software program. The mean fluorescence intensity (MFI) for each population was calculated.

### Infection assay

Ectocervical, endocervical and tUEC cells were seeded in 96-well tissue culture plates and incubated overnight in the respective antibiotic-free media. Bacteria were grown overnight on GC agar plates, suspended in D-PBS at ≅10^8^ cfu/mL in antibiotic-free medium. MOI (multiplicity of infection) was 100 bacteria per cell; aliquots of bacterial suspensions were diluted in D-PBS and plated at the time of infection for precise determination of bacterial starting inoculum. Cells were incubated with bacteria for 3 hours at 37°C in 5% CO_2_; to determine the number of intracellular bacteria, infected cells were washed four times with medium and treated with 200 μg/mL gentamicin for 1 hour at 37°C. After washing, cells were lysed by 1% saponin and plated. In parallel, to determine the growth rate of bacteria during the infection, bacteria without cells were incubated at 37°C in cell medium; after 3 hours the number of replicating bacteria was determined by serial dilution and plating. The bacterial colonies were monitored for piliation and Opa morphology by examination with a stereomicroscope.

For immunofluorescence analysis, ectocervical cells seeded on chamber slides were incubated with bacteria as described above. After incubation, wells were washed with PBS and fixed with 2% PFA for 20 min at room temperature. Subsequently, samples were blocked with 2% BSA for for 15 min and incubated with mouse polyclonal serum anti-OM (1:1000) for 1 h at room temperature. Wells were washed several times with PBS and incubated with goat anti-mouse Alexa Fluor 488 conjugated antibodies (Molecular Probes) and Alexa Fluor 568-conjugated phalloidin for 30 min at room temperature. Labeled samples were mounted with ProLong® Gold antifade reagent with DAPI and analyzed with Zeiss LSM710 confocal microscope.

## Competing interests

The authors declare that they have no competing interests.

## Authors’ contribution

RL designed the study, carried out experiments and analyses of the data and wrote the draft of the manuscript. BN performed the cloning and construction of knock-out mutant strain and supported the adhesion studies. EM contributed to FACS analysis. EC performed 2D electrophoresis. LS and RR participated in the planning of this study. MS participated in writing the manuscript. MP coordinated the study and assisted in writing the manuscript. All authors read and approved the final manuscript.
